# The Susceptibility to Diet-Induced Atherosclerosis Is Exacerbated with Aging in C57B1/6 Mice

**DOI:** 10.3390/biomedicines9050487

**Published:** 2021-04-29

**Authors:** Olivier Kamtchueng Simo, Hicham Berrougui, Tamas Fulop, Abdelouahed Khalil

**Affiliations:** 1 Program of Physiology, Faculty of Medicine and Health Sciences, University of Sherbrooke, Sherbrooke, QC J1H 1N1, Canada; Olivier.Kamtchueng.Simo@USherbrooke.ca (O.K.S.); Tamas.fulop@usherbrooke.ca (T.F.); 2 Department of Biology, Polydisciplinary Faculty, University Sultan Moulay Slimane, BP 592, Beni Mellal 23000, Morocco; hichamberg@gmail.com; 3 Department of Medicine, Faculty of Medicine and Health Sciences, University of Sherbrooke, Sherbrooke, QC J1H 4N4, Canada

**Keywords:** HDL, aging, atherogenic diet, reverse cholesterol transport

## Abstract

The anti-atherogenic activity of HDL is mainly due to their capacity to mediate reverse cholesterol transport (RCT). However, it is not clear to what extent this activity is affected by aging or pro-atherogenic conditions. Three and 24-month-old C57Bl/6 mice were fed an atherogenic diet (high fat, high cholesterol) for 12 weeks. The aged mice displayed a significant reduction in the capacity of HDL to mediate RCT (29.03%, *p* < 0.0006). Interestingly, the atherogenic diet significantly stimulated the RCT process in both young and aged mice (241% and 201%, respectively, *p* < 0.01). However, despite this, significant amounts of cholesterol accumulated in the aortas of mice fed an atherogenic diet as compared to regular chow. The accumulation of cholesterol was more marked in the aortas of aged mice (110% increase, *p* < 0.002). ABCA1 and ABCG1 protein expression on macrophages decreased significantly (52 to 37% reduction, *p* < 0.002), whereas their expression on hepatic cells increased significantly (up to 590% for ABCA1 and 116% for ABCG1, *p* < 0.002). On the other hand, SR-BI protein expression on hepatic cells decreased significantly (42.85%, *p* < 0.0001). ABCG5, ABCG8, and CYP7a protein expression on hepatic cells was also higher in mice fed an atherogenic diet. The increase was age-dependent for both ABCG5 and ABCG8. Our results suggest that the susceptibility to diet-induced atherosclerosis is exacerbated with aging and is a consequence of the dysregulation of the expression levels of membrane cholesterol transporters.

## 1. Introduction

Several clinical and epidemiological studies showed a negative correlation between plasma levels of high-density lipoproteins (HDL) and the risk of incidence of cardiovascular diseases [1,2].

The level of cholesterol in LDL and HDL depends on several factors, including the type of diet [3,4]. While the atherogenic diet can induce an increase in the level of LDL [5], some types of diets, might induce their beneficial effects by increasing the HDL levels [6]. However, increasing the HDL level is not always synonymous with cardiovascular protection [7]. For instance, the pharmacological inhibition of cholesteryl ester transfer protein (CETP) with Torcetrapib was associated with an increase in the number of cardiovascular events, despite a 72% increase in HDL cholesterol levels [8].

The static measurement of HDL cholesterol levels has inherent limitations as a metric of the functional effects of HDL in vivo [9]. In addition, reports of marked heterogeneity in the particle composition and biological properties of HDL pointed to a need for validated assays to measure HDL function [10]. The atheroprotective effects of HDL are attributed to the central role of HDL in anti-inflammatory, antioxidant, and reverse cholesterol transport (RCT) activities [11,12]. In fact, RCT is the main functional property of HDL [13]. During RCT, a series of reactions extract excess cholesterol from monocyte-derived macrophages in the sub-endothelial space and transport it back to the liver elimination [14,15]. The accumulation of cholesterol in macrophages leading to the formation of foam cells is a key step in the pathogenesis of atherosclerosis. This accumulation is the direct consequence of the alteration of the balance between the level of cholesterol transported at the peripheral level by LDL and that brought back from the peripheral cells to the liver, mainly via the RCT process [16].

Cholesterol efflux from macrophages is the first and most critical step of RCT. It is believed that, following hydrolysis of its esterified form, free cholesterol (FC) in macrophages and other cells is initially effluxed to lipid-poor/free apolipoprotein A-I (apo-AI), via ATP binding cassette transporter A1 (ABCA1). Nascent HDL formed by apoA-I lipidation via the ABCA1 transporter serve as an acceptor for additional free cholesterol from the ABCG1 transporter, which contributes to the formation of mature HDL. ABC membrane transporters play a significant role in cholesterol transport from macrophages to HDL and in the reduction of foam cell formation [17]. ABCA1 in hepatocytes also contributes to nascent HDL formation by mediating cholesterol efflux to apoA-I, whereas ABCG1 regulates both biliary cholesterol secretion and lipid accumulation. Alterations in the ABCA1 or ABCG1 expression might significantly affect the level of circulating HDL. It was reported that a lack of ABCG1 results in decreased plasma HDL cholesterol levels in mice fed a high cholesterol diet [18]. However, this is debatable since other studies were unsuccessful in demonstrating alterations in HDL levels in ABCG1-deficient mice fed a high-fat diet [19]. On the other hand, ABCG1 might be associated with changes in hepatic sterol biosynthesis in response to dietary fat [19].

Cholesterol homeostasis is also regulated by the ABCG5/ABCG8 heterodimer, which permits the transfer of cholesterol into bile [20,21]. ABCG5/G8 expression was also shown to increase 2-fold in mice fed a high cholesterol diet [22]. The expression levels of cholesterol membrane transporters can thus be influenced by diet, which makes it possible to maintain cholesterol homeostasis and prevents cholesterol accumulation in the arteries. However, it is unclear whether this effect is mediated by the modulation of RCT or whether this regulation might be affected in the presence of a risk factor for CVD such as aging. We hypothesize that, with age, there is an alteration in the level of expression of cholesterol membrane receptors and transporters, which could promote the development of atherosclerosis, especially in the presence of a proatherogenic diet.

The aim of the present study was thus to determine the effect of a high-fat high-cholesterol (HFHC) diet on RCT and its regulating transporters, and to determine how this process might change with age.

## 2. Materials and Methods

### 2.1. Chemicals

Polyethylene glycol (PEG), thioglycolate broth, Ethylenediaminetetraacetic acid disodium salt dihydrate (EDTA), hydrochloric acid (HCl), and 8-(4-chlorophenylthio)adenosine 3′:5′-cyclic monophosphate (cAMP) were purchased from Sigma-Aldrich (Oakville, ON, Canada). Sodium dodecyl sulfate (SDS), and Tween 20 were purchased from Fisher Scientific (Ottawa, ON, Canada). Ammonium persulfate and TEMED were purchased from Bio-Rad (Mississaugra, ON, Canada). Acrylamide/bis-acrylamide was purchased from Amresco (Solo, OH, USA). RIPA buffer was from Cell Signaling Technology (Boston, MA, USA). Western Lightning^®^ Plus-ECL and Cholesterol (1, 2- 3H (N)) (3H-cholesterol) were from Perkin Elmer (Guelph, ON, Canada). Lipoproteins measurement kit from purchased abcam (Cambridge, MA, Canada). Atherogenic diet (high-fat high-cholesterol diet; TD.02028) was purchased from Harlan Teklad (Indianapolis, IN, USA). Bovine serum albumin (BSA), fetal bovine serum (FBS), Dulbecco’s modified Eagle’s medium (DMEM), and penicillin/streptomycin were purchased from Wisent (St-Bruno, QC, Canada). J774 macrophage-like cells were obtained from American Type Culture Collection (ATCC, Manassas, VA, USA).

### 2.2. Animals and Diet

Twenty wild-type C57BL/6 mice were purchased from Charles River Laboratories (Sherbrooke, QC, Canada) and Twenty wild-type C57BL/6 aged mice were obtained from the Quebec Research Network on aging (Montreal, QC, Canada). Three and 20-month-old mice were submitted for 12 weeks to normal chow or a high-fat, high-cholesterol (HFHC) diet (15.75% fat, 1.25% cholesterol, and 0.5% sodium cholate). The mice body weights were monitored weekly. After 12 weeks of diet (chow or atherogenic diet), mice were fasted overnight, and blood was collected to assess the lipid profile (total cholesterol, HDL, and LDL cholesterol).

This study was approved by the Committee on the Ethics of Animal Experiments of the University of Sherbrooke (21 September 2017, Permit Number: 263-15BR). Animals were treated in accordance with Canadian council on animal care (CCAC). All efforts were made to minimize suffering. For all experiments, mice were first anesthetized with 1% isoflurane before being euthanized by CO_2_ inhalation, and death was confirmed by exsanguination.

### 2.3. Cholesterol Analysis

Total cholesterol and HDL-cholesterol levels, as well as LDL/VLDL levels were measured according to the manufacturer’s protocol, based on enzymatic colorimetric assay, with a commercial test kit from abcam (Cambridge, MA, Canada). The absorbance was read at 570 nm, using a Hitachi UH 5300 spectrophotometer.

### 2.4. Reverse Cholesterol Transport (RCT) Measurement

RCT measurement was determined as described previously by Ikhlef et al. [23]. In brief, J774 macrophages were radiolabeled with 4 μCi/mL of [^3^H]-cholesterol [24] and then suspended in DMEM, before being injected intraperitoneally into mice (~3.5 × 10^6^ dpm/mouse). Twenty mice were divided into 4 groups (diet and age). Blood samples were drawn at 0 h, 6 h, 24 h, and 48 h, and feces were collected during the 48 h period. After 48 h, mice were euthanized, and their livers were collected. [^3^H]-cholesterol levels in the plasma were determined by liquid scintillation counting, whereas [^3^H]-cholesterol in liver (50 μg), feces, and bile acids was extracted using the Folch method, as previously described [23]. The radioactivity in the samples was counted using a liquid scintillation counter. RCT was assessed by monitoring [^3^H]-cholesterol transfer from the labeled intraperitoneally injected macrophages to the plasma, liver, and finally to feces, and expressed as the percentage of radioactivity recovered. The plasma volume was estimated at 3.85% of body weight.

### 2.5. Isolation of Peritoneal-Elicited Macrophages

Primary peritoneal macrophages were obtained according to the method of Linton et al. [25]. In brief, macrophages were harvested five days after intraperitoneal injection of 4 mL of thioglycolate [25]. Macrophages were pelleted, re-suspended in DMEM supplemented with 5% FBS, and plated at a density of 10^5^ cells/cm^2^. These were allowed to adhere for 4 h and the non-adherent cells were removed by rinsing with PBS. Isolation of primary peritoneal macrophages was performed on mice other than those used for RCT measurements.

### 2.6. Extraction of Liver Membrane Proteins

Total protein from mice liver tissues were prepared using 20 mM tris buffer (pH: 7.5) containing 250 mM sucrose, 2 mM MgCl_2_, and a set of protease inhibitors. The membrane pellet was resuspended in a solution containing 80 mM NaCl, 2 mM CaCl_2_, 1% Triton X-100, 50 mM Tris-HCl, (pH: 8), and protease inhibitors.

### 2.7. Western Blot

Peritoneal macrophages and liver cells from wild-type mice fed with chow or HFHC diet were lysed in RIPA buffer. Protein extracts were analyzed on SDS-PAGE and were immunoblotted with ABCA1, ABCG1, ABCA5, ABCG8, or β-actin primary antibodies (Abcam, Cambridge, MA, USA), as previously described by Ikhlef et al. [26].

### 2.8. Measurement of Aortic Cholesterol Accumulation

Mice was euthanized and the aortic tissue was dissected between the iliac bifurcation and the heart, for the measurement of the level of the aortic cholesterol accumulation. The extraction of cholesterol was achieved as previously described [27]. The cholesterol level in aorta was measured according to the manufacturer’s protocol, using a commercially available test kit (ab65390 from abcam, Cambridge, MA, Canada). The absorbance was read at 570 nm using a Hitachi UH 5300 spectrophotometer.

### 2.9. Statistical Analysis

Statistical analyses were performed using the GraphPad Prism software, version 6.0 (GraphPad Prism-6 software^TM^, Inc., San Diego, CA, USA). Values are expressed as means ± SEM. Mean values were compared using the Student’s *t*-test. An ANOVA followed by Tukey multiple comparisons test was used to compare more than two groups. *p*-values less than or equal to 0.05 were considered significant.

## 3. Results

### 3.1. HFHC Diet on Lipid Profile

Male C57BL/6 young and aged mice were fed regular chow or an HFHC diet for 12 weeks. An HFHC diet is reported to cause a significant increase in plasma cholesterol and HDL cholesterol levels [28]. Our results showed that total cholesterol and HDL-c levels increased in both young and the aged mice, when they were fed the HFHC diet (41%, *p* < 0.001 and 23%, *p* < 0.005, respectively) (Table 1). This agreed with a previous study that reported that an HFHC diet causes a significant increase in plasma cholesterol and HDL cholesterol levels [28].

### 3.2. The Impact of Age and an HFHC Diet on RCT

**Figure 1:**
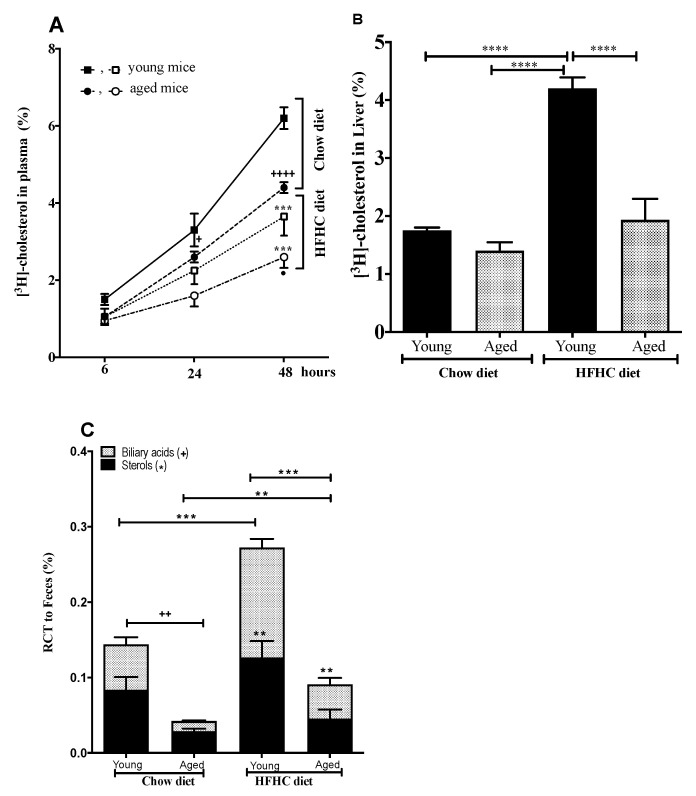
Effect of an atherogenic diet on RCT in young and aged mice. Young and aged mice (3- and 20-month-old mice, respectively) were fed chow or an HFHC diet for 12 weeks. [^3^H]-cholesterol-loaded J774 macrophages were intraperitoneally injected into the mice, and [^3^H]-cholesterol plasma levels were measured at 6, 24, and 48 h post-injection. Feces were collected continuously from 0 to 48 h post-injection. (**A**) Time course of ^3^H-cholesterol distribution in plasma. (**B**) ^3^H-cholesterol recovery in the liver after 48 h. (**C**) ^3^H-cholesterol recovery in the feces as sterols and biliary acid. Data are expressed as means ± SEM versus chow. *n* = 5 mice/group. ** *p* < 0.005, *** *p* < 0.001, **** *p* < 0.0001 versus chow diet, ^+^ *p* < 0.015, ^++^ *p* < 0.005 and ^++++^ *p* < 0.0003 young vs. aged mice.

Radiolabeled macrophages were injected intraperitoneally into young and aged C57BL/6 mice (respectively 3- and 24-month-old, respectively). [^3^H]-Cholesterol was measured in the plasma after 6, 24, and 48 h, as well as in the liver after 48 h, and in feces collected over a 48 h period. Unlike the young mice, plasma [^3^H]-cholesterol levels in the aged mice decreased by 29.03% after 48 h (*p* < 0.0006) (Figure 1A). There was no significant change in [^3^H]-cholesterol levels in the livers of either the young or the aged mice (Figure 1B). Fecal macrophage-derived [^3^H]-cholesterol levels were significantly lower in the aged mice than in the young mice (Figure 1C), with the aged mice displaying a 37.71% (*p* < 0.01) reduction in sterols and a 71.11% (*p* < 0.007) reduction in biliary acids.

To ascertain whether this age-related difference in the elimination of [^3^H]-cholesterol in the feces was modified under proatherogenic conditions, the young and aged mice were fed the HFHC diet for 12 weeks. Three RCT parameters were then measured. Surprisingly, both the young and aged mice fed the HFHC diet had lower plasma levels of [^3^H]-cholesterol than their counterparts who were fed chow (41% lower, *p* < 0.001) (Figure 1A). Interestingly, the age-related difference in the levels of plasma ^3^[H]-cholesterol at 24 and 48 h persisted even with the HFHC diet, albeit to a lower extent than that observed with chow (28.17% reduction in plasma [^3^H]-cholesterol levels in the aged mice at 48 h, *p* < 0.03) (Figure 1A).

On the other hand, the HFCH diet significantly increased [^3^H]-cholesterol levels in the livers of young mice (140% increase, *p* < 0.0001), while only a slight but insignificant increase was observed for the aged mice (Figure 1B). Fecal macrophage-derived [^3^H]-cholesterol elimination also significantly increased with the HFHC diet for both the young (201% increase, *p* < 0.001) and aged mice (241% increase, *p* < 0.01) (Figure 1C).

### 3.3. The HFHC Diet Increases Cholesterol Deposition in the Aortic Artery

**Figure 2:**
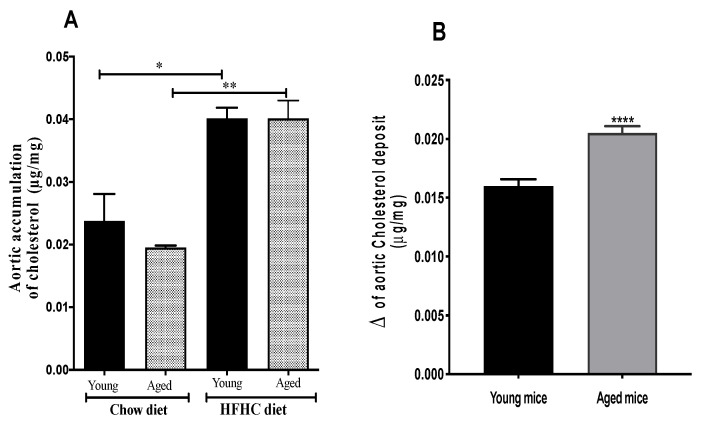
An HFHC diet increases the accumulation of cholesterol in the aorta. (**A**) Cholesterol levels in the aortas of young and aged mice fed an HFHC diet for 12 weeks compared to cholesterol levels in the aortas of young and aged mice fed chow for 12 weeks. (**B**) Difference in the cholesterol levels of the aortas of the young and aged mice fed an HFHC diet for 12 weeks. Data are expressed as means ± SEM (*n* =5 mice/group). * p < 0.05, ** p < 0.01, **** p < 0.0001 versus chow.

To determine whether changes in RCT with aging and with an atherogenic diet contribute to the acceleration of the atherosclerotic process, we measured cholesterol accumulation in the aorta. The HFHC diet significantly increased cholesterol deposition in the aortas of both young and aged mice (73.9%, *p* < 0.02, and 110%, *p* < 0.002, respectively), although there was no significant difference between the young and aged mice (Figure 2A). However, when compared to the chow diet, there was a significantly greater (*p* < 0.0001) deposition of cholesterol in the aortas of the aged mice than in the aortas of the young mice (Figure 2B).

### 3.4. The Expression of ABCA1 and ABCG1 on Macrophages Decreases with Age

**Figure 3:**
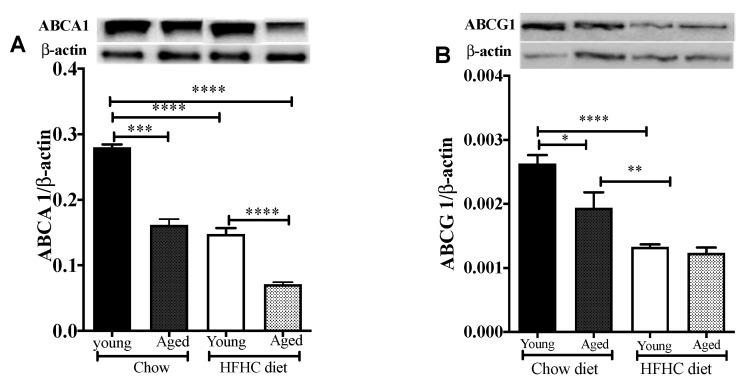
An HFHC diet affects ABCA1/ABCG1 protein expression on peritoneal macrophages. Young and aged mice were fed chow or an HFHC diet for 12 weeks. Peritoneal macrophages were obtained using the thioglycolate method and (**A**) ABCA1 and (**B**) ABCG1 protein expression was analyzed by Western blotting. Data are expressed as means ± SEM * p < 0.0213, ** p < 0.0091, *** p < 0.0003, **** p < 0.0001.

The efflux of cholesterol from macrophages to HDL is the first and rate-limiting step of the RCT process, which is initiated by an interaction of ABCA1 with lipid-free apoA1 (or preβ-HDL). We, thus, ascertained whether the decrease in [^3^H]-cholesterol plasma levels in aged mice compared to young mice was due to a difference in the expression levels of membrane cholesterol transporters on macrophages, particularly ABCA1 and ABCG1. Our results (Figure 3A,B) showed that there was a significant decrease in ABCA1 (42.29%, *p* < 0.0001) and ABCG1 (27.75%, *p* < 0.0001) protein levels in aged mice as compared to the young mice. Feeding the mice an HFHC diet significantly reduced the expression of the ABCA1 protein in both young and aged mice (46.9% and 52%, respectively, *p* < 0.0001) (Figure 3A). The age-related difference in ABCA1 protein expression levels was maintained when the mice were fed the HFHC diet, with the aged mice expressing significantly lower ABCA1 levels than the young mice (*p* < 0.0001) (Figure 3A). The same results were obtained for ABCG1 expression, when the mice were fed the HFHC diet, with a 50% (*p* < 0.0002) and 37% (*p* < 0.002) reduction for the young and aged mice, respectively (Figure 3B).

### 3.5. Aging and the HFHC Diet Upregulate the Expression of Liver Cholesterol Transporters

**Figure 4:**
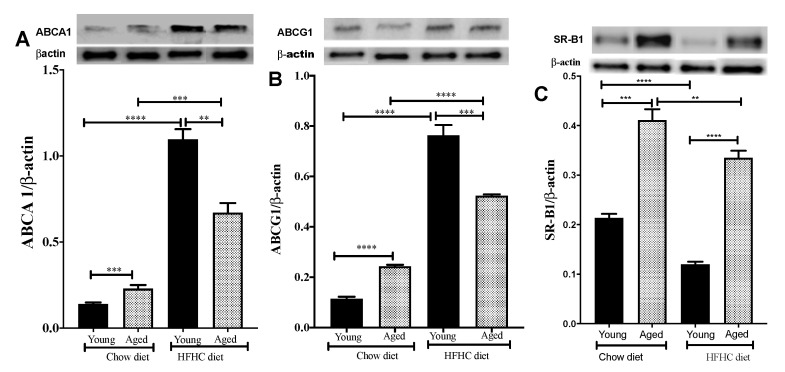
An HFHC diet affects ABCA1/ABCG1 and SR-BI protein expression by hepatic cells. Young and aged mice were fed chow or an HFHC diet for 12 weeks. They were then euthanized and (**A**) ABCA1, (**B**) ABCG1, and (**C**) SR-B1 protein expression in their livers was measured by Western blotting. Data are expressed as means ± SEM ** p < 0.0031, *** p < 0.0006, **** p < 0.0001 versus chow.

We investigated the impact of age and diet on the transfer of cholesterol from the plasma to the liver. We observed higher ABCA1 protein expression on the hepatic cells of aged mice (35.72% higher, *p* < 0.002) than those of young mice (Figure 4A). Higher levels of ABCG1 and SR-BI expression were also observed in the aged mice than in the young mice (91.93%, *p* < 0.001, and 95.23%, *p* < 0.0001, higher for ABCG1 and SR-BI, respectively; Figure 4B,C).

The HFHC diet also significantly increased the expression of ABCA1 (678%, *p* < 0.0001, and 191%, *p* < 0.002, higher in the young and aged mice, respectively; Figure 4A) and ABCG1 (590%, *p* < 0.001, and 116%, *p* < 0.002, higher in the young and aged mice, respectively; Figure 4B). On the other hand, the HFHC diet significantly reduced SR-BI expression in both the young (42.85% lower, *p* < 0.0001) and aged mice (19.51% lower, *p* < 0.02) (Figure 4C).

### 3.6. The HFHC Diet Upregulates ABCG5, ABCG8, and CYP7A1 Expression

**Figure 5:**
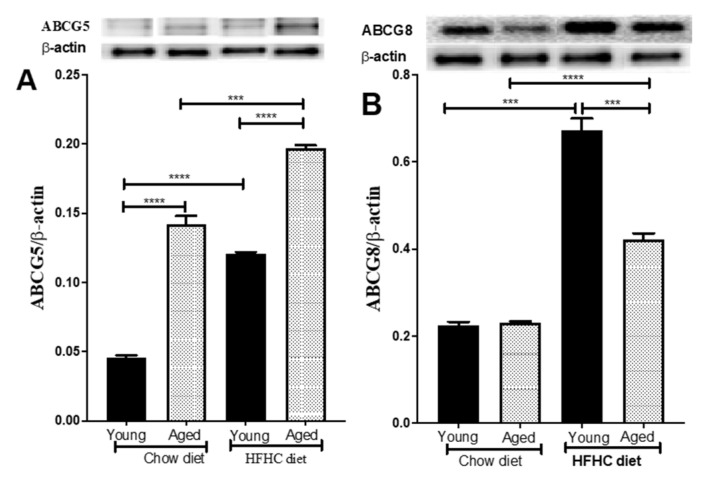
An HFHC diet upregulates ABCG5/ABCG8 protein expression by hepatic cells. Young and aged mice were fed chow or an HFHC diet for 12 weeks. They were then euthanized and (**A**) ABCG5 and (**B**) ABCG8 protein levels were measured in their livers by Western blotting. Data are expressed as means ± SEM. *** p < 0.0003, **** *p* < 0.0001 versus chow.

Cholesterol homeostasis in hepatic cells is also regulated by the activity of the ABCG5 and ABCG8 sterol transporters in the liver, which transfer excess dietary sterols from the liver to the intestine. We thus investigated the age-related expression of these two proteins and the role that the HFHC diet plays in regulating their expression. The aged mice expressed higher ABCG5 levels than the young mice (211% higher, *p* < 0.0001) (Figure 5A). However, there was no difference in ABCG8 expression (Figure 5B). Interestingly, the HFHC diet induced a significant upregulation of ABCG5 and AGCG8 (590%, and 116% higher, *p* < 0.0001, and 204% and 82%, higher, *p* < 0.001) for the young and aged mice, respectively (Figure 5A,B).

**Figure 6:**
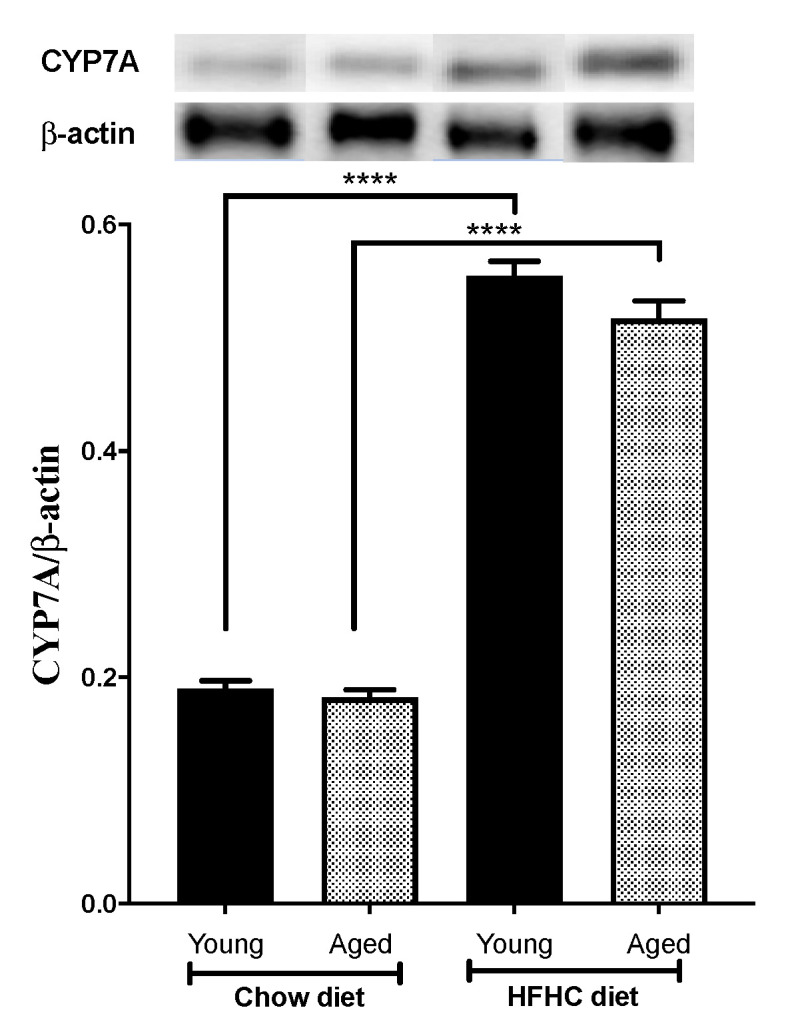
An HFHC diet upregulates CYP7A protein expression by hepatic cells. Young and aged mice were fed chow or an HFHC diet for 12 weeks. They were then euthanized and the CYP7A protein levels were measured in their livers by Western blotting. Data are expressed as means ± SEM, **** *p* < 0.0001 versus chow.

Cholesterol was eliminated from the liver into the bile either as cholesterol or as bile acids. Cholesterol was converted to 7-alpha-hydroxycholesterol by the cytochrome P450 7A1 (CYP7A1) enzyme, which was the first and rate-limiting step in bile acid synthesis. We, thus, investigated the age-related and diet-related expression levels of CYP7A1. Our results showed that CYP7A1 was equally expressed in both young and aged mice. (Figure 6). The HFHC diet induced an increase in the expression of CYP7A1 in both the young and old mice, albeit to a lower extent in the aged mice, but was not statistically significant (187%, *p* < 0.0001 versus 183%, *p* < 0.0001, respectively).

## 4. Discussion

The present study was designed to determine the impact of an atherogenic diet on RCT, on proteins that regulate RCT, and how this process changes with aging. Compared to the aged mice, the young C57BL/6 mice had a higher capacity to eliminate excess cholesterol via the RCT process. Previous in vitro studies showed that there is an age-related reduction in the capacity of HDL to mediate cholesterol efflux [29,30]. However, we were the first to show that there was an in vivo reduction in the capacity of HDL to mediate their main antiatherogenic activity, that is, to mediate RCT. It should be noted that the age-related reduction in RCT in our conditions was independent of HDL levels, which were not significantly different in the young and aged mice.

RCT is a multi-step process that depends on the capacity of HDL to extract cholesterol from cholesterol-loaded macrophages. It regulates cholesterol uptake by the liver and its excretion from the liver into the bile either directly or after conversion to bile acids. Although the efflux of cholesterol from macrophages is considered to be the first and rate-limiting step of RCT [31], other steps make significant contributions to this process [32]. ABCA1 and ABCG1 are key mediators in the initiation of cholesterol efflux from macrophages through their interactions with apoA-1 and lipid-poor HDL, respectively [33,34]. We did not observe a significant difference in plasma HDL concentrations between the young and aged mice. However, we did observe a significant decrease in the protein expression levels of ABCA1 and ABCG1 in macrophages from the aged mice, as compared to the young mice. Previous studies reported that the age-related decrease in the capacity of HDL to mediate cholesterol efflux can be explained by a reduction in HDL concentrations and in the capacity of HDL or lipid poor-apoA-1 to accept cholesterol [30,35,36]. Our results suggest that the predisposition of macrophages to transfer cholesterol via ABCA1 and ABCG1 carriers might also significantly affect the capacity of HDL to mediate cholesterol efflux from macrophages. ABCA1 expression is reduced in diabetes and is associated with a significant accumulation of cholesterol in macrophages [37]. Our results showed that aged mice fed an atherogenic diet accumulate more cholesterol in their aortas than young mice fed the same diet.

The delivery of macrophage-derived cholesterol to the liver is also a significant regulator of the RCT process and plays a major role in cholesterol homeostasis [38,39]. Hepatic SR-BI mediates the selective uptake of esterified cholesterol from mature HDL, and plays a critical role in RCT and atheroprotection [40]. Surprisingly, SR-BI expression was significantly higher in the hepatic cells of the aged mice, which contradicted the decrease in RCT observed in the aged mice as compared to the young mice. Nevertheless, overexpression of the SR-BI receptor is not synonymous with optimum functionality. It was shown that SR-BI also acts as a receptor for advanced glycation end (AGEs) product-modified proteins, which interfere with its role in RCT by inhibiting the selective uptake of HDL-associated esterified cholesterol [41,42]. AGEs accumulate with aging and can initiate a series of cellular events, including the alteration of SR-BI function, which contribute to the acceleration of the atherosclerosis process [42,43].

Like the SR-BI receptor, ABCG5 expression was also significantly higher in the aged mice than in the young mice, whereas there was no difference between the aged and young mice in terms of ABCG8 expression. The difference in the expression level of ABCG5 and ABCG8 in the aged mice is very surprising given that the expression of these two carriers is regulated by the LXRα pathway, which also regulates ABCG5 and ABCG8 mRNA expression [44]. On the other hand, since ABCG5 and ABCG8 form an obligate heterodimer to facilitate the secretion of hepatic sterols into bile [21,45], the increased expression of ABCG5 alone might not have a significant effect on cholesterol homeostasis. The expression of CYPPA1, which is the rate-limiting enzyme in the main pathway for the biosynthesis of bile acid from cholesterol was not different between young and aged mice. CYPPA1 expression is regulated by plasma cholesterol levels. While low plasma cholesterol levels cause a reduction in CYPA1 expression, increases in plasma cholesterol levels cause a significant upregulation of CYP7A1 expression [46,47]. Previous studies showed that the stimulation of CYP7A1 expression has a positive effect on RCT and plasma lipid level [46,48]. Our results also showed that HFHC diet induced an equivalent overexpression of CYPA1 in both young and aged mice, suggesting that the age-related reduction in RCT could not be explained by a difference in CYPA1 levels.

Feeding the mice an HFHC diet significantly stimulated RCT, as determined by the measurement of macrophage-derived [^3^H]-cholesterol levels in the feces. Radiolabeled-cholesterol levels in hepatic cells also increased when the mice were fed an atherogenic diet. These results agree with those obtained by Triguier et al. using hamsters [49]. However, our results were the first to investigate how age can affect the RCT process following the response to an atherogenic diet, in mice. Our results showed that stimulation of RCT by the HFHC diet was significantly higher in young mice than in aged mice. Although RCT increased significantly when the mice were fed a proatherogenic diet, this did not change cholesterol deposition in the aorta. Nevertheless, our results showed that ABCA1 and ABCG1 expression on macrophages was significantly decreased by the HFHC diet, which might explain the increase in cholesterol deposition in aorta, despite the significant stimulation of RCT. Indeed, as previously shown, the ability of ABCA1 and ABCG1 to promote cholesterol efflux from macrophages is an important pathway for limiting macrophage engorgement with cholesterol and the formation of foam cells [33].

Interestingly, the atherogenic diet has a different effect on ABCA1 and ABCG levels as a function of age, with aged mice displaying a significant reduction in ABCA1 and ABCG levels. These results suggest that the age-related reduction in RCT might be exacerbated under proatherogenic conditions. On the other hand, the expression levels of ABCA1, ABCG1, ABCG5, and ABCG8 increased significantly when the mice were fed the HFHC diet. The increase was significant for both age groups, although the increase was greater in the young mice than in the aged mice. Our results agree with those of others, who showed that an HFHC diet induces the overexpression of ABCA1, ABGC1, ABCG5, and ABCG8 in the liver [50].

RCT is a potential target for the prevention and regression of atherosclerotic vascular diseases [47]. An increase in RCT is associated with increased protection against the development of the atherosclerotic process [47,51]. However, our results showed that, despite stimulating RCT, the HFHC diet contributed to a significant accumulation of cholesterol in the aorta, suggesting that the increase in RCT caused by the atherogenic diet was insufficient to prevent the development of atherosclerosis. On the other hand, a cholesterol-rich diet might affect other mechanisms involved in the development of the atherosclerotic process, such as inflammatory macrophages or pro-inflammatory HDL [11,52,53]. Intriguingly, significantly more cholesterol was deposited in the aortic arteries of the aged mice after four months of the HFHC diet than in the aortic arteries of the young mice, suggesting that the proatherogenic effect of the HFHC diet might be more pronounced with advancing age. Our results agreed with those of Escola-Gil et al. who compared the effect of HFHC diets and high-saturated fatty acid and low cholesterol diets on RCT. Their study also showed that an HFHC diet causes an upregulation of ABCG1, ABCG5, and ABCG8 in the liver and an increase in RCT when the mice are fed an HFHC diet [50].

In the present study, we also focused on ABCA1 and ABCG1 expression levels in macrophages. Our results showed that, unlike their expression levels in the liver, ABCA1 and ABCG1 expression in macrophages was significantly reduced, which should normally have a negative impact on RCT. However, it must be noted that RCT is a dynamic process and that a decrease or alteration in cholesterol flux in one compartment might not automatically affect the total cholesterol removal. Conversely, our results showed that cholesterol efflux from macrophages plays an important role in the atherosclerotic process, since the alteration of cholesterol efflux from macrophages contributed to a significant increase in the deposition of cholesterol in the aorta, despite a significantly lower increase in RCT.

In summary, our results showed that feeding mice with an HFHC diet significantly stimulated RCT. This effect was more pronounced in the young mice than in the aged mice. The expression of cholesterol protein transporters in the liver (ABCA1, ABCG1, ABCG5, and ABCG8) was significantly upregulated in response to the HFHC diet, whereas the expression of ABCA1 and ABCG1 on macrophages was significantly downregulated. However, despite the fact that the HFHC diet caused a significant increase in RCT and in protein expression in the liver, the HFHC diet also contributed to a significant accumulation of cholesterol in the aorta, which was significantly exacerbated with age.

## 5. Study Limitations

One of the limitations of the present study was that the sex of mice was not considered in the analysis. Additionally, the effect of HFHC diet on the lipoprotein compositions (apoA-1, apoA-II, apoB48, and apoB100) and nutrient- and longevity-sensing proteins or genes were not investigated. These analyses would be of great interest to better understand the effect of diet and age on the metabolism of lipoproteins in general, on the distributions of different proteins and enzymes, and to define the nutritional and aging aspects of this animal model.

## Figures and Tables

**Table 1:** Effect of high-fat, high-cholesterol diet on total and HDL cholesterol.

	Young Mice		Aged Mice	
	Chow Diet	HF-HC Diet	Chow Diet	HF-HC Diet
Weight (g)	37.6 ± 2.58	34.8 ± 3.13 **	45.3 ± 7.97 ^++++^	43.03 ± 5.83 **** ^(^*^++++^*^)^
Total Cholesterol (mM)	2.43 ± 0.13	4.03 ± 0.17 **	2.3 ± 0.17	3.87 ± 0.038 **
HDL Cholesterol (mM)	2.06 ± 0.15	3.41 ± 0.10 ***	2.04 ± 0.12	2.61 ± 0.081 ** ^(^^+++)^
LDL/VLDL (mM)	0.31 ± 0.008	0.59 ± 0.031 **	0.21 ± 0.037	1.16 ± 0.035 *** ^(^^++)^

** *p* < 0.0083, *** *p* < 0.0003, **** *p* < 0.0001, for HF-HC diet compared to chow diet. ^++^ *p* < 0.014, ^+++^ *p* < 0.0006, ^++++^ *p* < 0.0001, comparison as a function of age for the same diet.
